# Ethylene enhances transcriptions of asparagine biosynthetic genes in soybean (*Glycine max* L. Merr) leaves

**DOI:** 10.1080/15592324.2023.2287883

**Published:** 2023-11-29

**Authors:** Gyeongik Ahn, Yeong Jun Ban, Gyeong-Im Shin, Song Yi Jeong, Ki Hun Park, Woe-Yeon Kim, Joon-Yung Cha

**Affiliations:** a Division of Applied Life Science (BK21four), IALS, RILS, and PBRRC, Gyeongsang National University, Jinju, Republic of Korea; b Herbal Medicine Resources Research Center, Korea Institute of Oriental Medicine, Naju, Republic of Korea

**Keywords:** Asn synthetase, asparagine biosynthesis, aspartate aminotransferase, ethylene, soybean

## Abstract

Soybean, a vital protein-rich crop, offers bioactivity that can mitigate various chronic human diseases. Nonetheless, soybean breeding poses a challenge due to the negative correlation between enhanced protein levels and overall productivity. Our previous studies demonstrated that applying gaseous phytohormone, ethylene, to soybean leaves significantly boosts the accumulation of free amino acids, particularly asparagine (Asn). Current studies also revealed that ethylene application to soybeans significantly enhanced both essential and non-essential amino acid contents in leaves and stems. Asn plays a crucial role in ammonia detoxification and reducing fatigue. However, the molecular evidence supporting this phenomenon remains elusive. This study explores the molecular mechanisms behind enhanced Asn accumulation in ethylene-treated soybean leaves. Transcriptional analysis revealed that ethylene treatments to soybean leaves enhance the transcriptional levels of key genes involved in Asn biosynthesis, such as aspartate aminotransferase (AspAT) and Asn synthetase (ASN), which aligns with our previous observations of elevated Asn levels. These findings shed light on the role of ethylene in upregulating Asn biosynthetic genes, subsequently enhancing Asn concentrations. This molecular insight into amino acid metabolism regulation provides valuable knowledge for the metabolic farming of crops, especially in elevating nutraceutical ingredients with non-genetic modification (GM) approach for improved protein content.

## Introduction

Plants serve as a rich source of natural nutrients that meet human dietary needs and provide health benefits.^1^ Medicinal and herbal plants, in particular, have been integral to traditional healthcare routines worldwide.^2,3^ These plants contain essential compounds that drive the development of advanced pharmaceutical products.^4^ The human healthcare market, with a specific focus on nutraceuticals, has experienced substantial growth due to the renewed recognition of the effectiveness of natural resources derived from plants.^5^ Within the realm of botanical diversity, plants synthesize a wide array of primary and secondary metabolites through intricate biochemical pathways.^6–8^ These metabolites exhibit various therapeutic and biological properties, including antimicrobial, antioxidant, anti-inflammatory, anti-cancer, and anti-diabetic effects.^9–12^ However, production from plants often falls short of industrial demand.^13^

Among the diverse array of metabolites, amino acids play a pivotal role in maintaining normal growth and development.^14^ Amino acids serve as the fundamental building blocks of proteins, which are essential nutrients for living organisms.^15^ These amino acids are classified as either essential or nonessential, based on the body’s capacity for direct synthesis.^15^ They are abundant in protein-rich foods such as meat, fish, and even plants like soybean (*Glycine max* L. Merr).^15,16^ Soybean cultivated for more than 5,000 years is an important crop for food and oil production and its seeds are major sources of plant-derived proteins.^17^ Moreover, recent studies showed that consumption of soybean-based proteins reduces various chronic diseases, including cardiovascular disease, women’s menopausal symptoms, cancer, osteoporosis, and abdominal body fat.^16^ However, high protein content in soybean is often negatively correlated with key agronomic traits, including productivity, making it challenging to select soybean varieties with both high protein content and productivity.^17^

Interestingly, the leaves instead of soybean seeds have emerged as promising nutraceutical sources, attributed to their various biological effects, including anti-obesity, antidiabetic, and anti-inflammatory properties.^18,19^ Extracts from soybean leaves contain a rich in bioactive compounds, including phenolic compounds, flavonoids, and soyasaponin derivates.^20,21^ Our previous studies have demonstrated that application of gaseous phytohormone, ethylene, leads to a substantial increase in the levels of phytoestrogens such as daidzein, genistein, malonyl-daidzein, and malonyl-genistin.^22,23^ Most recently, our gas chromatography-mass spectrometry (GC-MS) analysis showed that ethylene treatments to soybean leaves increase the amount of free amino acids by five-fold, particularly asparagine (Asn).^24^ Asn is not only vital for protein synthesis, but also plays a crucial role in ammonia detoxification within cells, consequently reducing muscular fatigue.^25^ Despite ethylene’s ability to enhance Asn levels in soybean leaves, the molecular evidence supporting this phenomenon remained incomplete. This study bridges that gap by revealing that ethylene treatments lead to increased transcriptional levels of Asn biosynthetic genes, a facet previously unexplored.

## Materials and methods

### Plant materials and ethylene treatments

Soybeans (*Glycine max* L. Merr) were cultured in soil pots within a greenhouse under environmental condition as previously described^24^ until reaching the V6 growth stages. Sixty soybean pots were then transferred to an air-sealed chamber with controlled humidity (70%) and temperature (35°C). Ethylene treatment was applied at a concentration of 2,500 µg/g, and the ethylene concentration inside of the chamber was maintained by monitoring with a portable sensor (COSMOS, *P*-3160, Japan). For the measurement of essential and nonessential free amino acid contents, ethylene was treated for 24 hrs, and samples were harvested separately for leaves, stems, and roots. To assess the transcription of Asn biosynthetic genes, leaf samples were harvested at 12, 24, 48, and 72 hrs after ethylene treatment and immediately frozen at −78°C. All samples from the pots were randomly mixed and harvested.

### Quantification of free amino acids using HPLC

Free amino acid contents were conducted as previously described.^24^ Briefly, the milled samples (100 mg) were extracted in deionized water at 60°C for 1 h, and protein/peptides were precipitated using 10% (v/v) sulfosalicylic acid. After centrifugation, the supernatant was filtered, and the solvent was removed using a vacuum evaporator at 45°C. The concentrate was dissolved in 0.2 M lithium citrate buffer (pH 2.2) and filtered through a 0.45 μm filter. About 20 μL samples were injected into a Biochrom + 30 system (Biochrom Ltd.) equipped with a high-pressure PEEK (Poly. Ethyl Ketone) column packed with Ultropac8 cation exchange resin. Amino acids were eluted with varying conditions (temp., ionic strength, and pH of the lithium citrate buffer). Amino acids were detected using the reaction of ninhydrin with amino acids that formed colored compounds and were then photometrically detected at 440 nm and 570 nm for primary amines and secondary amines, respectively. Amino acids were calibrated and quantified using known standards (Sigma-Aldrich).

### RNA isolation, cDNA synthesis, and real-time quantitative RT-PCR (qRT-PCR)

Total RNA was extracted from ethylene-treated leaf samples using HiGene Total RNA Prep Kit (Biofact), and cDNA was prepared from total RNA (3 μg) using RevertAid First Strand cDNA Synthesis Kit (Thermo Scientific). qRT-PCR was performed using soybean gene-specific primers (Supplementary Table S1) and TOPreal qPCR PreMIX Kit (Enzynomics) in CFX96 Touch Real-Time PCR detection system (Bio-Rad). The qRT-PCR conditions were as follows: 95°C for 5 min; 45 cycles of 95°C for 15 s, 55°C for 15 s, and 72°C for 30 s; followed by 72°C for 5 min. Melting curves were analyzed at the end of PCR with a temperature range of 65‒95°C in 0.5°C increments for 5 s. The relative expression was calculated using the comparative cycle threshold (ΔΔCt) method, and normalized to the expression of the soybean housekeeping gene, *β-tubulin* (*β-TUB*).

## Results and discussion

**Figure 1.**
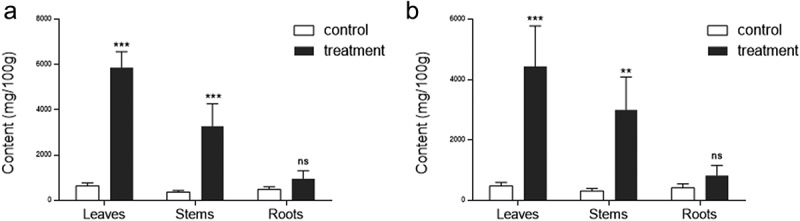
Effects of ethylene treatment on free amino acid contents in leaves, stems, and roots of soybean. (a) total essential amino acids and (b) total non-essential amino acids. Asterisks indicate statistically significant differences between control and ethylene treatment (Student’s t-test, ***P < .001; **P < .01; ns, not significant).

We verified the remarkable increase in free amino acid contents induced by ethylene treatment, as we previously observed,^24^ by categorizing it into leaves, stems, and roots of soybean. For both essential and non-essential free amino acids, ethylene treatment resulted in a nine-fold increase in leaves and stems, and an approximately two-fold increase (but not significant compared to control) in roots (Figure 1a, b). This finding aligns with our previous research indicating significant elevation in free amino acid contents in soybean leaves due to ethylene treatment. Consequently, the ethylene application, particularly on leaves that constitute the majority of harvested soybean yield, suggests a convenient approach for maximizing the production of valuable substances such as free amino acids through metabolic farming.

**Figure 2.**
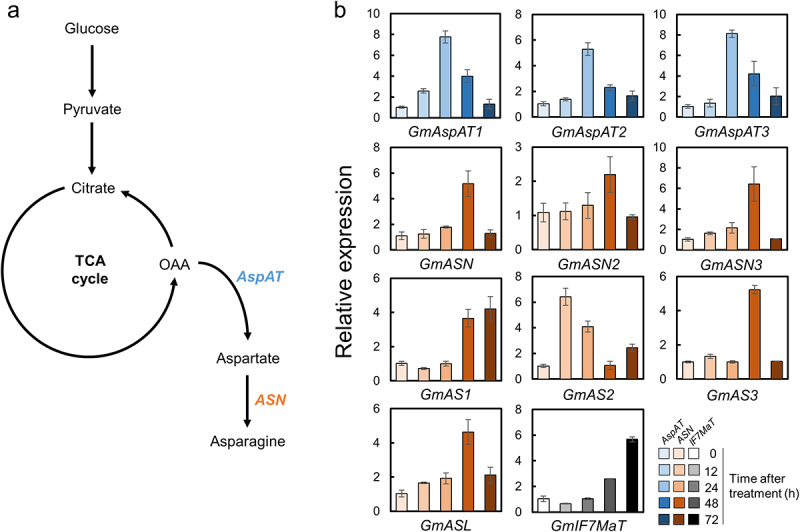
Relative expression of asparagine biosynthetic genes in soybean exposed to ethylene. (a) the schematic diagram of asparagine biosynthesis. OAA, oxaloacetate; AspAT, aspartate aminotransferase; ASN, asparagine synthetase. (b) the mRNA levels of AspAT isoforms (blue), ASN isoforms (orange), and IF7GT (gray) were analyzed by qRT-PCR and normalized to the levels of ubiquitin as an internal control. Data represent the mean±SE with three independent biological replicates.

To decipher precise molecular evidence of what ethylene application enhances Asn contents in soybean leaves, we checked the transcriptional levels of Asn biosynthetic genes in soybean leaves exposed to ethylene. Ethylene was applied to soybean plants at V6 growth stage for 12, 24, 48, and 72 h within a controlled chamber. Asn is synthesized through the coordinated action of two essential enzymes: aspartate (Asp) aminotransferase (AspAT/AAT, EC:2.6.1.1) and Asn synthetase (ASN/AS, EC:6.3.5.4). This synthesis occurs from oxaloacetate (OAA), with AspAT transferring the α-amino group from glutamate (Glu) to OAA to form Asp, and ASN catalyzing the amidation reaction, transferring the amide group from glutamine (Gln) to Asp to generate Asn (Figure 2a).^26^ The soybean genome database reveals the existence of five genes encoding AspAT (*GmAspAT1*, 100811514; *GmAspAT2*, 100814593; *GmAspAT3*, 547509; *GmAspAT4*, 547792; *GmAspAT5*, 547813) and seven genes encoding ASN (*GmASN*, 547877; *GmASN2*, 100798318; *GmASN3*, 100788806; *GmAS1*, 547895; *GmAS2*, 547894; *GmAS3*, 732660; *GmASL*, 100797022). Our findings showed that the transcriptional levels of three *GmAspAT* genes were notably upregulated, peaking at 24 h post-ethylene treatment. Additionally, most of the *GmASN* genes exhibited increased transcription levels, with a peak at 48 h, except for *GmAS2*, which peaked at 12 h after ethylene treatment (Figure 2b). We also examined the transcriptional level of the isoflavonoid biosynthetic gene isoflavone 7-*O*-glucoside-6-*O*-malonyltransferase (*IF7MaT*), previously reported to be upregulated by ethephon (an ethylene donor) exposure^22^, and observed its peak upregulation at 72 h following ethylene treatment. AspAT plays a significant role in amino acid synthesis using changes in carbon skeletons derived from the TCA cycle and reversibly catalyzes the transfer of amino groups between aspartate and glutamate.^26,27^ In Arabidopsis, five *AspAT* genes are localized in various subcellular compartments, such as mitochondria, cytoplasm, plastid, and peroxisome.^26,27^ Similarly, soybean *AspAT* genes exhibit diverse subcellular localization; *GmAspAT1* and *GmAspAT4* are found in chloroplasts, *GmAspAT2* and *GmAspAT5* in mitochondria, and *GmAspAT3* in the cytoplasm. We observed that the transcriptional levels of these differentially localized *GmAspAT1*, *2*, and *3* were enhanced, increasing up to 5–6 fold at 24 h after ethylene treatment compared to levels at 0 h (Figure 2b). Consequently, Asp concentrations in soybean leaves increased up to 8.5-fold following ethylene treatment.^24^ It is worth noting that ethylene biosynthesis is intricately regulated by a network of aminotransferases. Previous studies involving 1-aminocyclopropane-1-carboxylic acid (ACC), an ethylene precursor, demonstrated a reduction in Asp levels in *Brassica napus* seedlings, leading to diminished root and shoot development.^28,29^ Our results suggest that ethylene treatments can differently regulate Asp levels depending on the plant species. Asp is subsequently converted into Asn through an ATP-dependent amidation reaction catalyzed by ASN.^26^ In Arabidopsis, three ASN genes have been identified, with AtASN1 sharing 77% sequence identity with both AtASN2 and AtASN3, which exhibit 91% sequence identity with each other. Soybean ASN is divided into two classes: class I, consisting of GmASN, GmASN2, GmAS1, GmAS2, and GmASL, with 89–99% identity among them, and class II, including GmASN3 and GmAS3, with 96% sequence identity. Intriguingly, most of the *ASN* transcripts in soybeans were upregulated at 48 h after ethylene application, aligning with the peak expression of all AspAT transcripts at 24 h (Figure 2b). This induction of *ASN* genes resulted in an impressive 25.3-fold increase in Asn levels compared to control conditions.^24^ Collectively, our investigation reveals that ethylene application sequentially upregulates key genes involved in the Asn biosynthetic pathway, including *AspAT*s and *ASN*s, leading to a substantial increase in Asn concentrations within soybean leaves.

## Conclusions

This study addresses a crucial challenge in enhancing the nutraceutical components of soybeans without resorting to genetic modification. Soybean, a valuable source of protein with well-documented health benefits, exhibits a significant boost in the essential amino acid Asn levels following ethylene treatment. Our research delves into the intricate molecular mechanisms governing this phenomenon, shedding light on the transcriptional upregulation of pivotal genes involved in Asn biosynthesis. This gene activation coincides with the observed rise in Asn levels following ethylene treatment. This molecular understanding of amino acid metabolism regulation carries broad implications, particularly for metabolic farming strategies aiming to enhance crop nutraceutical value without genetic modification, all while optimizing protein content and overall productivity. This research contributes to the evolving field of plant biochemistry, offering the potential for more efficient and sustainable crop production methods.

## Supplementary Material

Supplemental.docx
