# Supplementary material for: Ethylene enhances transcriptions of asparagine biosynthetic genes in soybean (Glycine max L. Merr) leaves
Source: Plant Signal Behav. 2023 Nov 29;18(1):2287883. doi: 10.1080/15592324.2023.2287883 (PMC10761183; doi:10.1080/15592324.2023.2287883)
Supplement: Supplemental.docx [file KPSB_A_2287883_SM9844.docx]

**Supplemental online material for**

Ethylene enhances transcriptions of asparagine biosynthetic genes in soybean (*Glycine max* L. Merr) leaves

Supplemental material contains one Supplemental table.

Supplementary Table 1. Primer sequences used in this study.

| Gene | Accession No. | Primer sequence |
| --- | --- | --- |
| *GmAspAT1* | 100811514 | For: 5'-CTACTACTTGATCTCATCATGGCT-3’ |
|  |  | Rev: 5'-TGCCTTCAAACCGAGAAACG-3’ |
| *GmAspAT2* | 100814593 | For: 5’-TTGGTGGGACCATGTAAGGC-3’ |
|  |  | Rev: 5'-GGAAGCGTTTGACTCCTCCAA-3’ |
| *GmAspAT3* | 547509 | For: 5'-ACTTTTCCATTACGCACGCA-3’ |
|  |  | Rev: 5'-TAAGCGACAGTTACCCCGAG-3’ |
| *GmASN* | 547877 | For: 5'-GGAGTTGCCATATAGAGCTAG-3’ |
|  |  | Rev: 5'-ATAGCCTTGCCTGAATAAACT-3’ |
| *GmASN2* | 100798318 | For: 5’-GGAGTTGCCATATAGAGCTAG-3’ |
|  |  | Rev: 5'-AAGATGTGGATGGCACAAAGA-3’ |
| *GmASN3* | 100788806 | For: 5'-CCCCTTTAAGGGCCCATAATA-3’ |
|  |  | Rev: 5'-TCCAAACAGTTCAAGACCTAC-3’ |
| *GmAS1* | 547895 | For: 5'-GAGTTGCCATATAGAGCTTGT-3’ |
|  |  | Rev: 5'-TTCCCTGGATAAACTACAAAGC-3’ |
| *GmAS2* | 547894 | For: 5'-CCATCCAGGGCTAATACAAAG-3’ |
|  |  | Rev: 5'-CGCAACACTTTTTAGAAAGCC-3’ |
| *GmAS3* | 732660 | For: 5'-GCTGTGGAATGGGATGCA-3’ |
|  |  | Rev: 5'-TCCCCACTAGACAGTTGTTGC-3’ |
| *GmASL* | 100797022 | For: 5'-CCAGGGATAATATAAAGCTGTG-3’ |
|  |  | Rev: 5'-AAGACTTCAAATCACAGCATTC-3’ |
| *GmIF7MaT* | NM001250831 | For: 5'-CCCTCTCTTCAAACCTCTCAG-3’ |
|  |  | Rev: 5'-TGGTGGCTTGTTATTCCTATC-3’ |
| *Gmβ-TUB* | X60216 | For: 5'-GCTGATGGTGTATGGTTTTG-3’ |
|  |  | Rev: 5'-TTGCCCAGGGAAACG-3’ |
